# Remote drain inspection framework using the convolutional neural network and re-configurable robot Raptor

**DOI:** 10.1038/s41598-021-01170-0

**Published:** 2021-11-17

**Authors:** Lee Ming Jun Melvin, Rajesh Elara Mohan, Archana Semwal, Povendhan Palanisamy, Karthikeyan Elangovan, Braulio Félix Gómez, Balakrishnan Ramalingam, Dylan Ng Terntzer

**Affiliations:** 1 Engineering Product Development Pillar, Singapore University of Technology and Design (SUTD), Singapore, 487372 Singapore; 2 LionsBot International Pte. Ltd., #03-02, 11 Changi South Street 3, Singapore, 486122 Singapore

**Keywords:** Engineering, Electrical and electronic engineering

## Abstract

Drain blockage is a crucial problem in the urban environment. It heavily affects the ecosystem and human health. Hence, routine drain inspection is essential for urban environment. Manual drain inspection is a tedious task and prone to accidents and water-borne diseases. This work presents a drain inspection framework using convolutional neural network (CNN) based object detection algorithm and in house developed reconfigurable teleoperated robot called ‘Raptor’. The CNN based object detection model was trained using a transfer learning scheme with our custom drain-blocking objects data-set. The efficiency of the trained CNN algorithm and drain inspection robot Raptor was evaluated through various real-time drain inspection field trial. The experimental results indicate that our trained object detection algorithm has detect and classified the drain blocking objects with 91.42% accuracy for both offline and online test images and is able to process 18 frames per second (FPS). Further, the maneuverability of the robot was evaluated from various open and closed drain environment. The field trial results ensure that the robot maneuverability was stable, and its mapping and localization is also accurate in a complex drain environment.

## Introduction

Drains are a critical piece of infrastructure in the urban environment. They are responsible for transporting either rainwater, wastewater, or a combination of both. A routine drain inspection is an essential service in metropolitan cities. There are over 600,000 km of public sewer lines in Germany. In Singapore, it is estimated that the public net of the drain is extended to over 1.2 million kilometers. Globally, two million tonnes of plastic litters enter into rivers and oceans each year through drains. It heavily affects our ecosystem and creates a lot of water-borne diseases in human society. Apart from that, water blockage in the drain due to bushes, trash, mud creates critical infrastructure damage in heavy rainfall^1^. Hence, routine drain inspection is mandated in urban regions. It will help mitigate water-borne and vector-borne diseases, saving human lives and safe critical infrastructure from floods. Human visual inspection is a commonly used method for drain inspection by sewer and waste management companies. This method includes identifying the trash and silt accumulation inside the drain and monitoring the intrusion of bushes and tree roots. For drain inspection, they use various inspection tools like a snake, plunger, wire brushes, and pipe camera. However, this is labor-intensive, time-consuming, difficult to monitor multiple locations simultaneously or over extended periods, and prone to high risk of accidents in drains of enormous diameter. Further, workforce shortage has been a crucial issue to sewer and waste management companies over the past decade. The shortage in the workforce due to health problems, working in a complex environment of sewer networks, and low wages is another critical challenge faced by drain management companies. As a consequence, automation of drain inspection is the best course of action.

In literature, various algorithms and tools are reported to automate the drain inspection task. Typically, closed-circuit television (CCTV)^2–7^, sonar^8^, laser scanner^9^, infrared^10^, and robots^11–16^are commonly used inspection tools, and computer vision techniques are used for an alternate of human visual inspection. The computer vision techniques have been categorized into traditional computer vision and learning-based computer vision techniques. In contrast with traditional computer vision, learning-based computer vision increases the prediction performance and greater accuracy in tasks such as image classification^17,18^, semantic segmentation^19,20^, object detection^21,22^, and Simultaneous Localization and Mapping (SLAM)^5^. In addition, this approach often requires less expert analysis and provides superior flexibility as frameworks can be re-trained using a custom data set.

Deep convolutional neural network (DCNN) are widely used in learning-based computer vision techniques for drain inspection instead of traditional computer vision algorithms. The DCNN framework is directly or remotely bridged with closed-circuit television (CCTV) or a robotics vision pipeline to perform the automated visual drain inspection task. In^2^, Ruwan et al. automate the storm–water pipe inspection using DCNN. The authors tested the trained DCNN models on a held-out validation set created from CCTV video and achieved an average precision of 0.77. In another study, Jack et al. introduced automated detection of sewer pipe defects by utilizing CCTV inspection videos. A Faster Region-based convolutional neural network (Faster R-CNN) is constructed, and several hyper-parameters are adjusted to study the influential factors. Higher detection accuracy is achieved by adjusting filter size and stride values, resulting in mAP of 83%^3^. Saeed et al. proposed an automated approach for inspection and condition assessment of sewer pipelines using computer vision techniques. This approach identifies a region of interest (ROI) using hidden Markov models (HMM) to extract abnormal frames from sewer CCTV videos and uses convolutional neural networks (CNN) to detect the defects and classify them in data sets from CCTV inspection reports^4^. In^5^, the authors proposed a framework for tracking multiple sewer defects in CCTV videos based on defect detection and metric learning. The experiments show that the framework can track sewer defects in CCTV videos and achieve a F1 score of 57.4%. The research by Syed et al. presented a framework for automated sewer defect classification and recognition of defect location in CCTV inspection videos based on deep learning. The AlexNet model was trained and validated by extracting the images from CCTV videos. The highest accuracy recorded was 96.33%^6^. Dang et al. suggested a deep learning-based automated sewer defect classification framework for the collected images by CCTV videos. The authors addressed the imbalanced data problem using XGBoost, LightGBM, and misclassification cost customization. The model was successful in achieving classification accuracy of 95.7%^7^. The above studies suggested that CCTV and DL for drain inspection. However, CCTV based inspection has lot of practical issues in deployment and maintenance. Deployment and maintaining CCTV for long-range drain networks is a challenging task. More cameras are required to cover a more extensive area of the drainage system and accurately pinpoint the exact location of the defect in the drain. This further increases the cost of CCTV drain inspection.

Robot-assisted drain inspection is an alternative method for CCTV inspection. The robot is equipped with a high-power vision system, and remote monitoring system built with a high-level visual inspection algorithm. PIRAT, a sewer inspection robot, was introduced by Kirkham et al. to measure geometry and detect defects. The authors employed a laser scanner and sonar scanner to measure geometry and for flooded sewers, respectively. A combination of machine vision and neural network techniques was used to identify, classify automatically, and rate pipe defects^11^. Another semi-autonomous wheeled tethered robot named KARO was reported in^12^. The robot was equipped with sophisticated multi-sensors for smart sensor-based sewage inspection. In^13^, the authors presented KANTARO, a fully autonomous, untethered robot that can access straight and bendy pipes using a specific mechanism known as the “naSIR Mechanism.” A small 2D laser scanner is used with a fisheye camera to assess the pipe state and fault detection. KURT is a six-wheeled untethered autonomous robot that can fit through 600 mm diameter pipes^14^. MAKRO is an untethered autonomous worm-shaped wheel with multiple segments, and an autonomous engine for drainage system navigation^15^. Tarantula is a self-configurable drain mapping robot that can adapt and morph according to a drain that has multiple level shifts^16,23,24^.

However, the robot-assisted inspection also has some practical shortcomings. Generally, robot-based inspection uses fixed morphology robots, which are mostly design for sewer pipe or tunnel inspection and hard to fit for inspecting uneven complex open and closed drain environments. As a result, limited efficiency is achieved during the inspection tasks. Reconfigurable robots are more flexible than fixed-size robots. These robots can adopt dynamic environment changes and can access the constricted environment of the drain. It has been used widely for various automation applications including cleaning indoor and outdoor^25,26^, inspecting the built environment^27,28^, ship hull inspection, aircraft structural inspection^29^, etc. These robots are developed with an inherent capability to change their morphology to perform another motion such as climbing^30^, rolling^31^, flying^32^ and floating^33^.

Conclusively, the above literature survey indicates that many researchers have tried to automate the drain inspection using CCTV, robot, and DCNN algorithms. However, they only focus on drain structural or construction defects but do not address common drain-blocking objects such as accumulated trash, bushes, and silt. Also, these studies have used CCTV or fixed morphology robot-based tools for automating drain inspection. Fixed morphology robots have some practical limitations for use in the dynamic environment of open and closed drains. Reconfigurable robots are more flexible than fixed-size robots. To the best of our knowledge, there is no direct case study using AI powered reconfigurable robots for detecting the common drain-blocking objects. This work presents DCNN based drain-blocking object detection using our in-house developed reconfigurable robot Raptor to bridge the research gap and overcome the shortcomings.

This paper is organized as follows; Section “Introduction” has an introduction, motivation, and literature review; Sect. “Overview of proposed system” presents the overview of the proposed system. Next, Sect. Experimental setup and results consists of the experimental setup, results, and discussion. Finally, Sect. Conclusion presents the conclusion of this research work.

## Overview of proposed system

**Figure 1:**
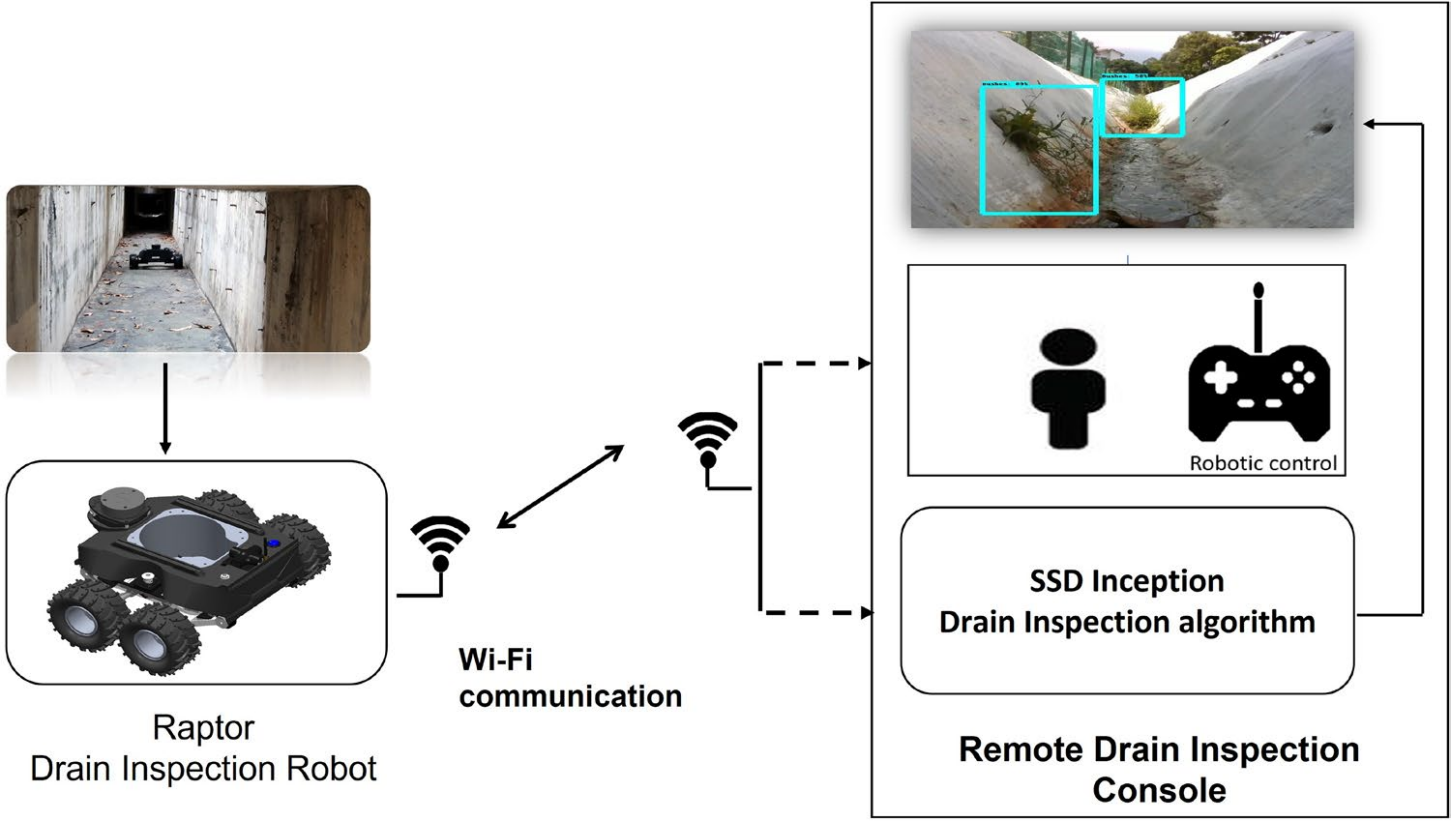
Overview of the drain inspection framework.

Figure 1 shows the overview of the drain inspection framework. Here, a deep learning-based object detection framework was used to perform the visual drain inspection task from our in-house developed drain inspection robot Raptor collected images. The details of drain inspection algorithm and robot architecture are described as follows.

### SSD Inception object detection framework

SSD Inception is an object detection framework that is used for the drain inspection task. It’s a lightweight framework and widely used in mobile applications and onboard object detection in robotics. In our drain inspection application, SSD automatically detects the bushes, trash, and silt from raptor transferred images. Typically, VGG-16 or MobileNet is used as a feature extractor for SSD. In our implementation, VGG-16 is replaced by the Inception feature extractor to achieve optimal detection accuracy and faster detection. The detail of Inception and SSD functionality are described as follows.

**Table 1:** Comparison of the Inception models^34^.

Models	MACC	COMP	ADD	DIV	Activations	Parameters
Inception v1	1.5G	15.98M	3.28M	3.23M	15.71M	6.62M
Inception v2	2.2G	8.48M	12.55M	3.25M	18.03M	11.19M
Inception v3	5.72G	16.53M	25.94M	8.97M	41.33M	23.83M
Inception v4	12.27G	21.87M	53.42M	15.09M	72.56M	42.71M
Inception-ResNet v2	13.18G	31.57M	38.81M	25.06M	117.8M	55.97M

MACC = Multiply and accumulate, COMP = comparison, ADD = addition, DIV = division.

#### Inception

In our proposed framework Inception v3 was utilised after evaluating its performance and computational complexity. A brief discussion of Inception v3, Inception v4 and Inception-ResNet v2 is mentioned below. As seen in Table 1 a 2 times jump in parameters is observed when comparing Inception v3 with Inception v4^35^ and Inception-ResNet v2^35^. Szegedy et al. further improved upon the existing Inception v3 architecture by making changes in the stem of both pure Inception v4 and Inception ResNet v2 network, introducing the reduction modules and standardising the inception modules to be more uniform to reduce complexity and improve performance at the same time. Szegedy et al. was able to produce a gain of 0.4–1.3% over Inception v3 architecture on the ILSVRC 2012 validation set using various crops. In the experimental results, replacing the Inception v3 feature extractor with Inception v4 and Inception-Resnet v2 did not yield the additional gain of 0.4–1.3% as produced by Szegedy et al.. The results can be seen in Table 4 and further elaboration is done in the experimental section.

**Figure 2:**
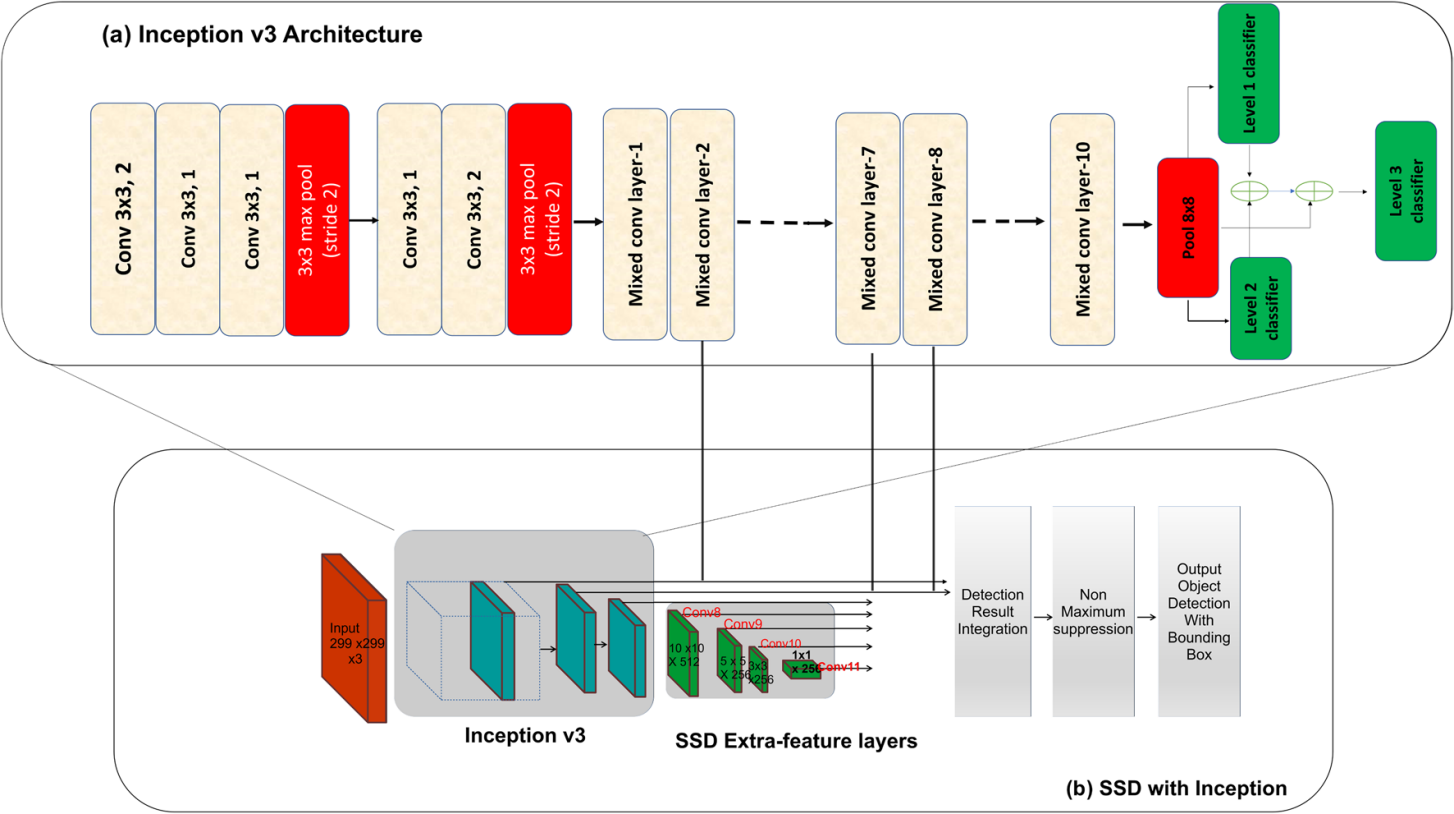
SSD Inception v3 object detection framework.

Figure 2a shows the neural network architecture of the Inception framework. Inception v3^36^ is an updated version of the Inception v1^37^ algorithm and uses batch normalization, dropping dropout, and removed the local response normalization function. In contrast with other feature extractor, the Inception algorithms use wider networks with filters of different kernel sizes in each layer, making it translation and scale-invariant. In Inception v3, $5\times 5$ convolution is factorized by the two $3\times 3$ convolutions and $7\times 7$ convolutions is replaced by series of $3\times 3$ convolutions. This increases the performance of the architecture and reduces the computational time (typically $5\times 5$ convolution is 2.78 more expensive than $3\times 3$ convolution). Further, to reduce the representational bottleneck issue, the feature banks of the module were expanded instead of making it deeper. Here, the algorithm factorizes convolutions filter size where $3\times 3$ convolutions function is converted into $1\times 3$ then followed by $3\times 1$ convolution. This factorize process takes 33% lesser computation compared to $3\times 3$ convolution and prevents the loss of information that is caused when a deeper architecture is used. A $17 \times 17 \times 768$ feature map is extracted from the Inception v3 framework and used as the input into the SSD framework to perform the object detection task. It uses the outputs of ‘Mixed_2d’, ’Mixed_7’, ‘Mixed_8’ of InceptionV3 framework.

#### SSD

Figure 2b shows the functional block diagram of SSD^38^ based object detection framework. It runs on top of the Inception v3 feature extractor algorithm. SSD can execute object detection tasks using a single neural network structure by converting the classification and localization steps of the object detection task into a regression problem. SSD comprises of the pyramid structured numerous auxiliary convolutional layers on top of Inception CNN layers to perform the object detection and classification task, which are utilized to extract the feature map at different resolutions and enable the network recognize varied sizes of objects. SSD detects and locates objects using six feature maps. Here, the first two sets are obtained from the Inception module and the remaining feature maps ($10 \times 10, 5 \times 5, 3 \times 3, 1 \times 1$) produced using SSD auxiliary convolution layers. SSD also outputs the bounding box coordinates and class type, as well as the classification confidence level, using bounding box predictors. The bounding box predictors use the default anchor boxes (bounding boxes) with different aspect ratios and scales. These bounding boxes have a fixed size, implying that their dimensions are chosen from a list of predefined values. The fixed size anchor boxes are tiled on generated feature map in a convolution manner and perform the one prediction per default box as well as the per-class scores that indicate the presence of a class instance in each of those boxes. In the end, Non-Maximum Suppression (NMS) is applied to remove the overlapping boxes and keep the highest-rated bounding boxes.

### Overview of drain inspection robot Raptor

**Figure 3:**
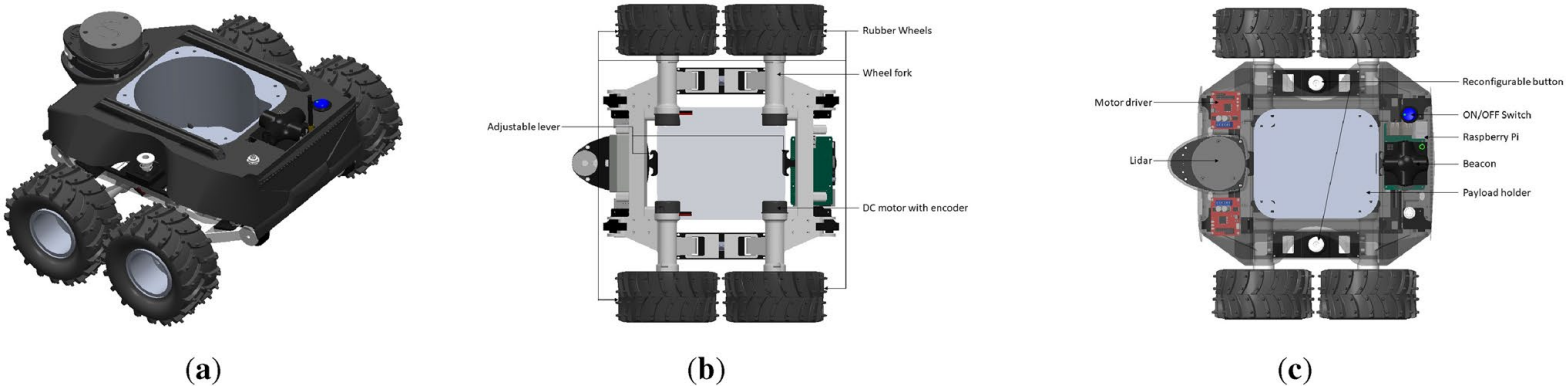
Raptor mechanical overview.

Structural overview of the Raptor robot is shown in Fig. 3. The structural frame and cover of Raptor are 3D printed with nylon to have greater tensile strength. The overall weight of the robot is 2.45 kg which can achieve a vertical gradient of 20$^{\circ }$–25$^{\circ }$. The detailed technical specifications of the platform are given in the Table 2. With a compact dimension of $390\times 350\times 200$ mm, the platform can navigate through constrained drain sizes.

The robot’s locomotion was accomplished by two adjustable dual-wheel forks assembled under the front and back sides of the chassis. These forks hold the DC motors for the motor to connect with the wheels. As shown in Fig. 3a, b, the Raptor design is centered around the payload holder to maintain the center of gravity in the middle of the robot. Most of the electronics components are mounted on the center body of the robot, where a mounting structure was provided. It also allows the assembly of electronic components and batteries. Furthermore, some additional mounting structure is added to fix the Lidar, camera, obstacle avoidance sensors in front of the robot.

**Table 2:** Technical specifications of Raptor.

Description	Specification
Platform weight	2.45 kg
Payload	1.6 kg
Dimension	$390\times 350\times 200$ mm
Environmental	3D printed nylon for prototyping
Ground clearance	98 mm stowed, 150 mm unstowed
Maximum linear velocity	0.22 m/s
Maximum angular velocity	0.85 rad/s
Maximum gradient	20, 25
Maximum side gradient	18, 20
Traverse terrain	Tested on short grassland and rough concrete terrain

### System architecture

**Figure 4:**
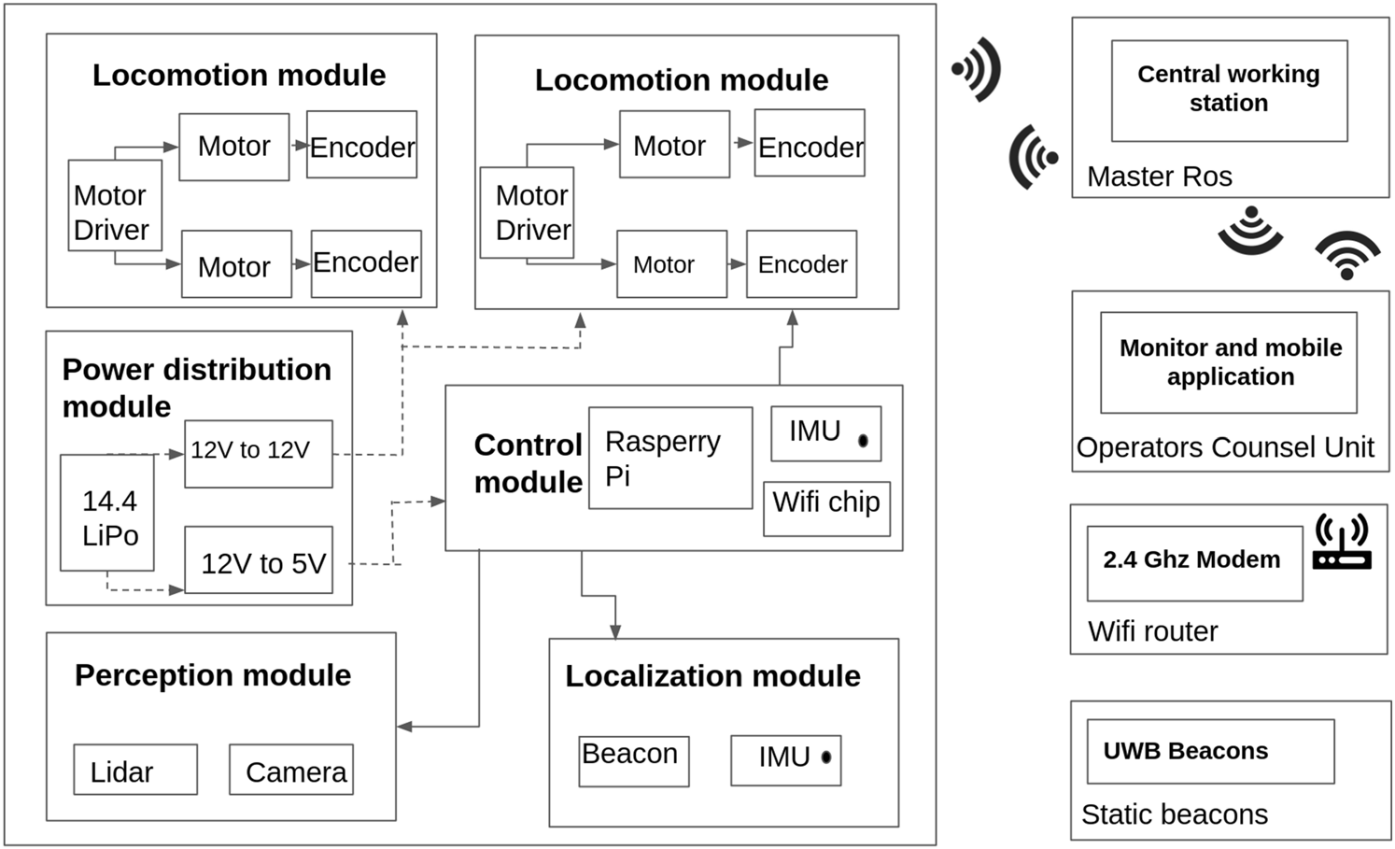
System architecture.

The high-level system architecture for Raptor is built using the robot operating system (ROS) framework. The system consists of a mobile platform—Raptor, master control station mobile control Unit for operators. Here, all the communication happens through a WiFi router with TCP/IP protocol. Figure 4 shows the high-level and the low-level control block diagram with all the associated modules.

#### Locomotion module

A four-wheel-drive system configuration powered the locomotion of the robot. It is powered by a 12 V DC metal gear motor that ensures the wheel pulls the load on all terrains. In addition, the fork holding wheels to the chassis is provided with extra reinforcement to withstand the direct impulse forces from the ground. These DC motors have a stall torque of 7.8 kg cm. With the air-filled rubber tires, the wheels ensure maximum friction, cushion effect, and ride height for the platform.

#### Re-configurable module

**Figure 5:**
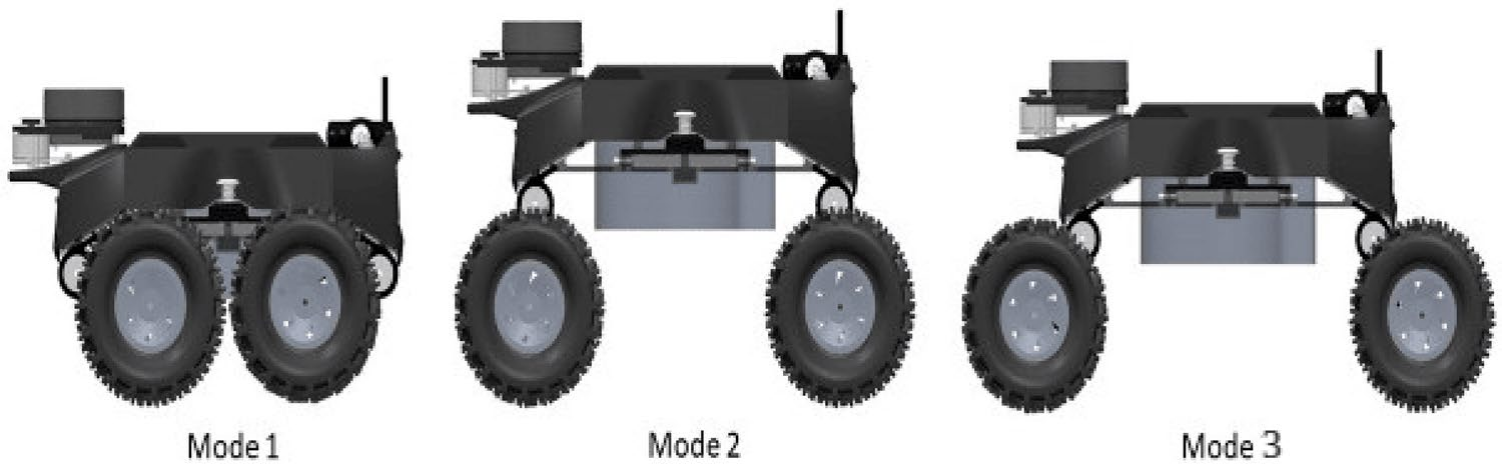
Raptor three different re-configuration modes.

Our Raptor has 3 modes of manual re-configuration as shown in Fig. 5. Here, Mode 1 is fully folded and used to carry a heavy payload weight of around 1.6 kg with a high ground grip and traverse all types of terrain. Mode 2 is halfway open for carrying a heavy payload and traversing all types of terrain, specifically for locomote over the obstacles and stagnant water area, and Mode 3 is used for high-speed and surveillance purposes. The reconfiguration function has been controlled by manually triggering the push button switch attached to both sides of the robot. This will release the folded wheel and move to Mode 1, Mode 2, and Mode 3, respectively.

#### Control module and path tracking

The on-board control unit for Raptor was built using RaspberryPi, which is capable of processing heavy data from Camera, Lidar, Inertial measurement units (IMU) and beacon modules. The control system takes linear velocity, and angular input commands directly from the operator and outputs individual motor speed signals using the inverse kinematics method. Each motor is pre-programmed to operate independently based on the specific input commands. For instance, if the input serial command is to turn the robot right, the two wheels on the inside radius spin in the opposite direction but proportional to that on the outer side. Each Roboclaw motor driver controls 2 DC brushless motors with 7.5 A till 15 A peak through pulse width modulation (PWM) signals from Raspberry Pi microcontroller.

A 2D Lidar sensor, RP-Lidar with rotation speed of 10 Hz and with sampling rate of 8000 points per second, is used for perceiving the environment for mapping and localization. This high sampling rate improves the mapping accuracy and map density. To map an unknown environment, hector slam algorithm is used to perform simultaneous localization and mapping (SLAM).

#### Power distribution module

The power distribution module includes a 4-cell Lithium-ion battery, 14.4 V 2800 mA h. Through a toggle switch connected to the main battery, two voltage regulators provide a steady supply of 12 V for the motor driver and 5 V for the Raspberry embedded computing device. The four motors are individually powered through the two motors drivers, with all other sensors powered through the Raspberry Pi.

#### Tele operation with collision avoidance safety layer

**Figure 6:**
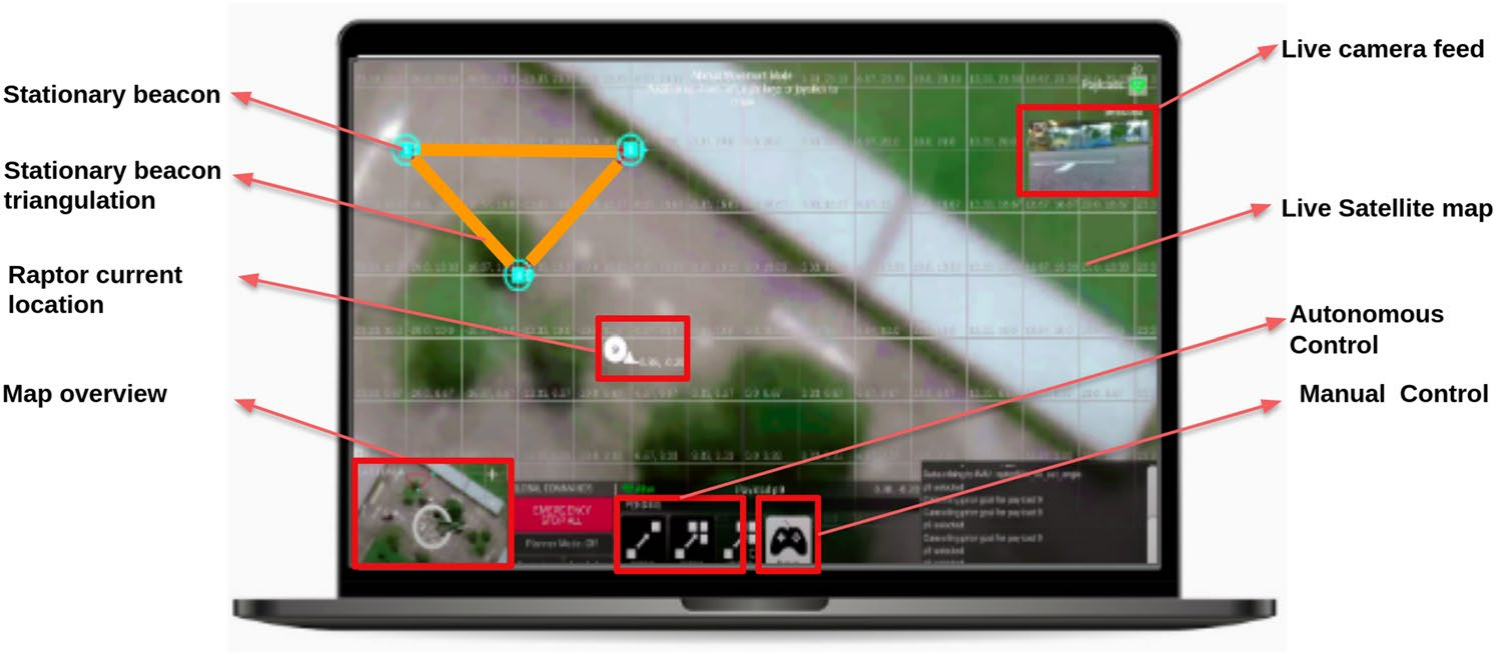
Operators control unit.

Raptor is designed to be teleoperated by users through mobile or laptop applications. This multi-device compatible graphical user interface (GUI) application is developed using Unity. The communication between robots and control devices happens via the message queuing telemetry transport (MQTT) bridge through ROS. ROS messages are serialized by JSON for MQTT communication and deserialized back for ROS messages in the communication protocol. Figure 6 shows the GUI console which has the provision for monitoring the video feeds and robot control buttons for basic operations like linear forward and backward movement, pivot rotation.

With Lidar sensor data as input, an additional safety layer is added to avoid unintentional obstacle collision due to the carelessness of the operator. In order to tackle this scenario, ROS architecture is configured with DWA local planner to compute multiple possible trajectories and to choose the cost-efficient path to maneuver around static and dynamic obstacles.

## Experimental setup and results

This section describes the experimental results of the drain inspection framework. The experiment was conducted in four phases: collecting training images, training the drain inspection framework, evaluating the trained model on both offline and real-time field test, and comparing the trained model with other object detection frameworks and existing works.

### Training images collection and labelling

**Figure 7:**
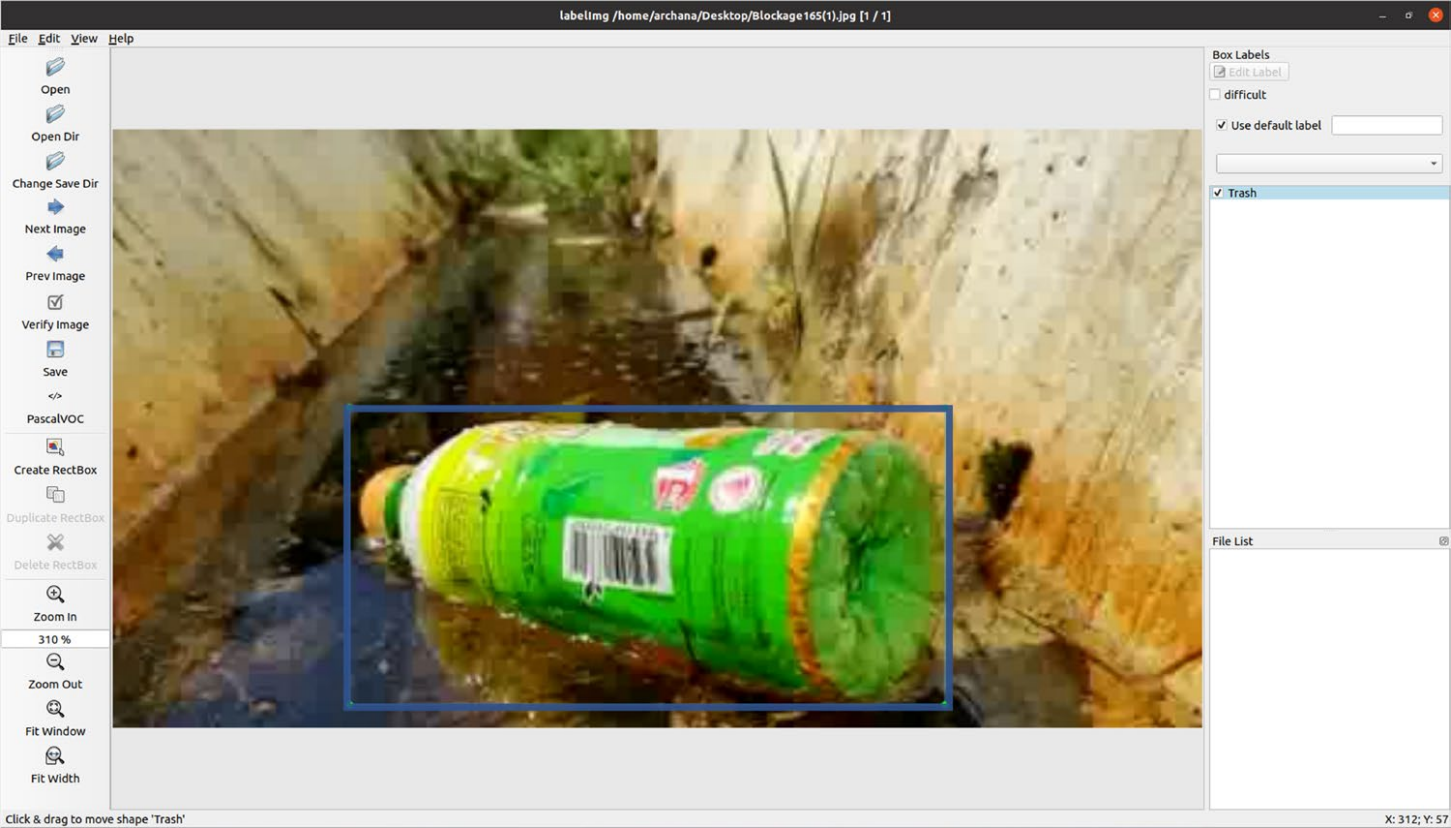
Bounding Box annotation tool.

**Figure 8:**
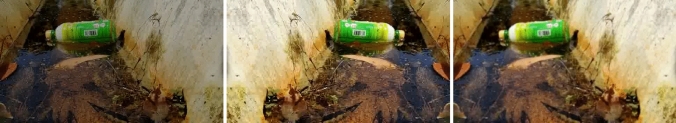
Data-augmentation of one input image.

The training images collection process involve collecting the drain blocking objects images from different sources. The data-set is composed of common drain blocking objects and categorised into three main classes such as trashes (dry leaves, plastics bottles, metals cans, paper, and trash), bushes and silt accumulation. Our data set consists of 4500 images gathered from open and closed drainage’s in Singapore. Before labeling, the collected images are resized into $640 \times 480$ and fed into “LabelImg” GUI tool. It’s an open-sourced bounding box annotations tool and is used to mark the objects bounding region. Thereafter, the bounding box data augmentation function is applied on the labeled images^39^. Figures 7 and 8 shows the bounding box annotation method and bounding box data augmentation results of one input image.

The data augmentation function converts the single image into multiple images with bonding box marking and uses the contrast adjustment, scaling, rotation, and flipping function on inputted images. The bounding box data augmentation function enlarges the data-set size, helps in controlling the over-fitting issues in training time, and enhances the detection framework learning rate.

#### Training and testing

##### Training hardware and software details

The drain inspection algorithm was developed in TensorFlow 1.15 open source machine learning platform running on Ubuntu 18.04.5 LTS version. The model was trained and tested via Google Colaboratary using the Intel(R) Xeon(R) CPU @ 2.00 GHz, 12.69 GB random access memory (RAM) and Nvidia Tesla P100 server graphics card (3584 CUDA cores).

##### Parameter configuration

Transfer learning method was used to train the drain inspection model. It’s more adaptable, allowing pre-trained models to be used directly as feature extraction. In our experiment, Imagenet dataset trained weight files was used to train algorithm under transfer learning scheme. Under the transfer learning scheme, the detection model was trained with 100,000 epochs and using a batch size of 16, the RMSProp algorithm of 0.9 momentum and an initial learning rate of 0.004, respectively. The image data set is evaluated using the k-fold cross-validation process. Images are divided into k groups in this technique, with $k-1$ being used to train the network. The one pair that remains is used for testing. 10-fold cross-validation approach is used in the proposed study. The visuals of the experimental data derived from the model are very accurate.

#### Evaluation metrics

The efficiency of our trained model was evaluated in both offline and real-time test scenarios. Standard metrics were used to evaluate both detection and classification model performance. Here, accuracy, precision, recall and $F_{measure}$ (1)–(4) were used to evaluate the model and confusion matrix was constructed to find the variables *tp* (true positives), *fp* (false positives), *tn* (true negatives) and *fn* (false negatives).

1 $$\begin{aligned}&Accuracy (Acc) = \frac{tp + tn}{tp + fp + tn + fn} \end{aligned}$$

2 $$\begin{aligned}&Precision (Prec) = \frac{tp}{tp + fp} \end{aligned}$$

3 $$\begin{aligned}&Recall (Rec) = \frac{tp}{tp + fn} \end{aligned}$$

4 $$\begin{aligned}&F_{measure} (F_{1})= \frac{2 \times precision \times recall}{precision + recall} \end{aligned}$$

### Offline test

**Figure 9:**
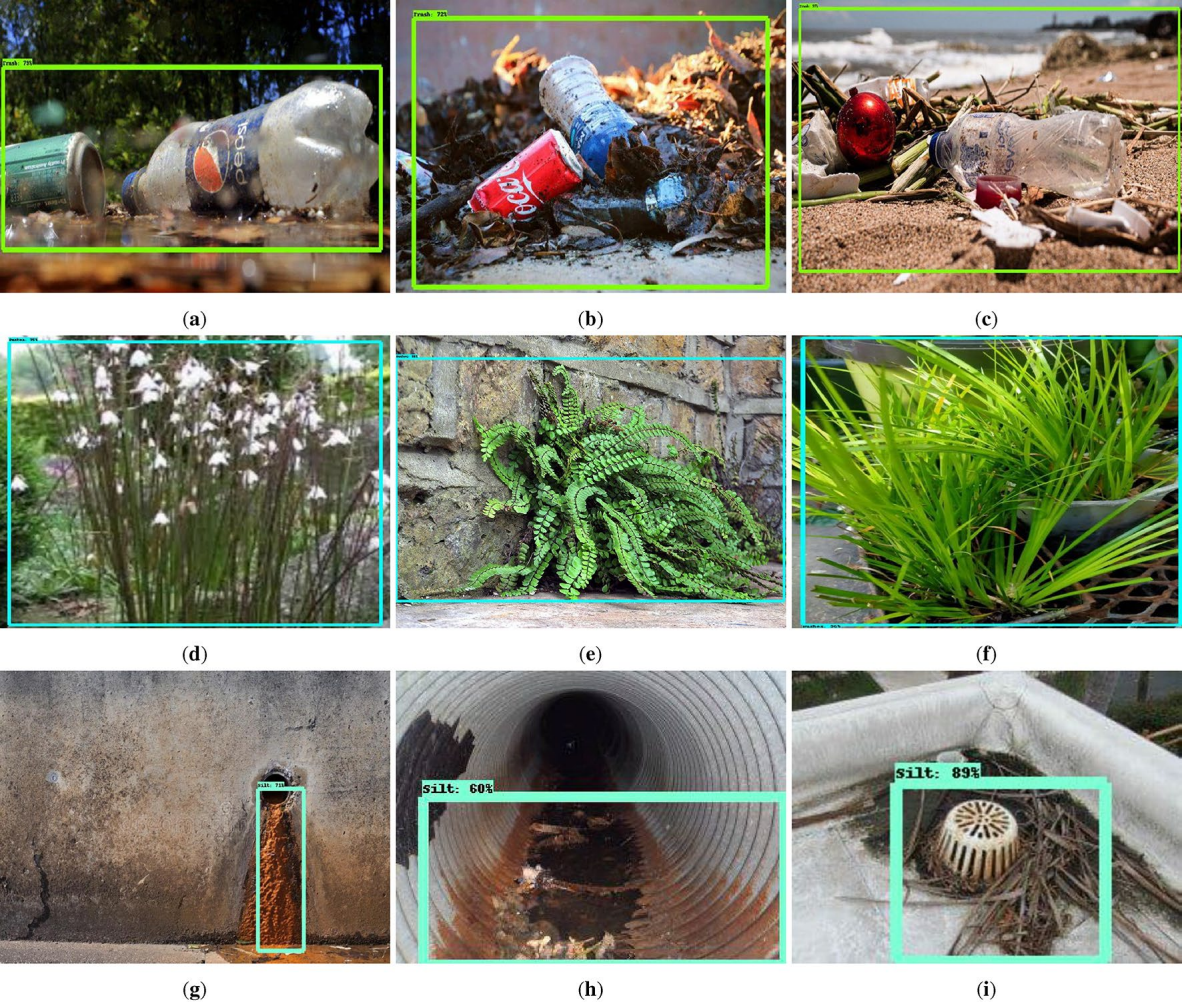
Offline test-web collected images trash, bushes and silt detection.

**Figure 10:**
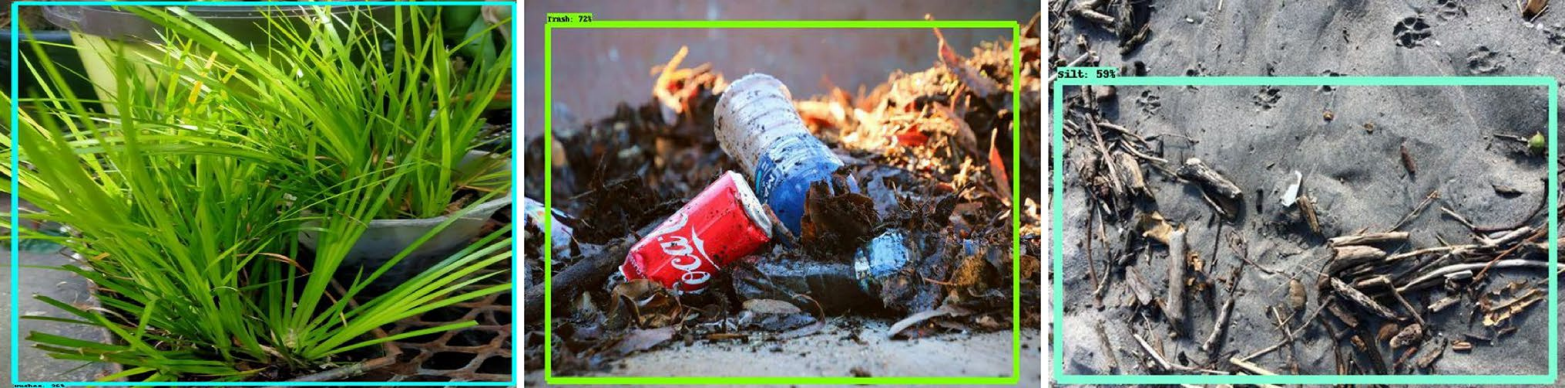
Taco data-set detection result.

**Figure 11:**
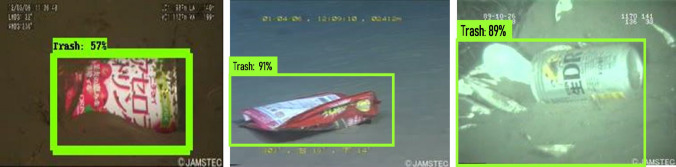
Deep sea data-set detection result.

**Figure 12:**
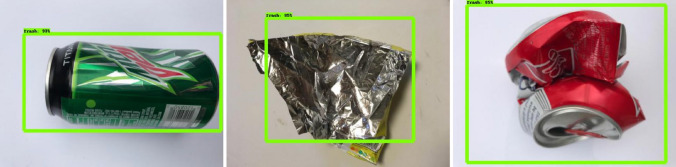
Trashnet data-set detection result.

The offline experiment was performed using web collected images and standard data-sets images including trashnet^40^, deep-sea waste^41^ and taco^42^. Here, the three data-sets are composed of various classes of litters collected from diverse environment. In each dataset, 50 images were selected for offline test composed of metal, plastic cans, polythene covers and paper trashes. Figures 9, 10, 11 and 12 show the drain inspection framework offline experimental results. Here, the algorithm detect and classify trash category with average of 85–94% confidence level, bushes detected with 88–96% confidence level and accumulated silt are detected with 80–89% confidence level respectively.

In this analysis, we observe that the algorithm detect most of the visible objects with higher confidence level. The miss detection, false classification, detection with lower confidence level happened only when inference is done on decomposed objects or partially occluded objects. The offline experimental results shows that the trained model is able to detect the drain blocking objects in diverse environment with better accuracy. Hence, the model can be used for detecting the drain blocking objects in diverse drain environment.

### Experiments with Raptor in real-time drain inspection field trial

Three different drain environments were selected for the real-time field test: closed drain, semi-closed drain, and open-drain located near the SUTD campus. These drains are used for rainwater collection, 1–3 ft. depth. The experiment was carried out in two lighting conditions (day and night) and two different climate days (after heavy rainfall and summer days). In the first field trial, the raptor robot operation efficiency was tested in terms of maneuverability. In the next step, trash, bushes and silt detection was evaluated from Raptor captured field images.

**Figure 13:**
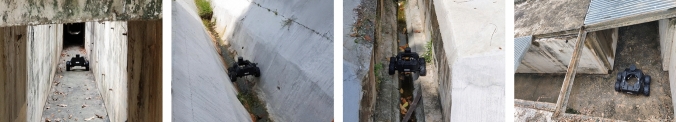
Raptor maneuverability test in different drain terrains.

**Figure 14:**
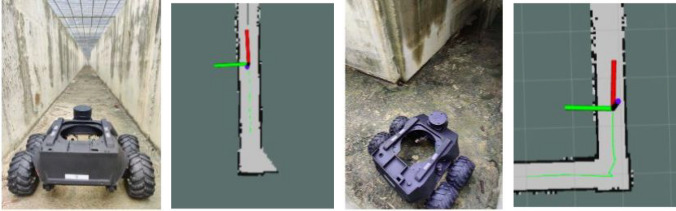
Mapping and path tracking results of Raptor.

Figure 13 shows the Raptor robot maneuverability test in various drain environments. In this experiment, the robot is deployed in an unknown drain environment with no prior preparation of drain map. Here, the drains are of different structures and sizes. There are areas inside the drain with long open tunnels of uneven concrete terrain with many L-shaped turns, narrow curves, and level changes. Hector slam algorithm was used to map the drain environment. The SLAM algorithm localizes the robot position with respect to the generated map simultaneously. Figure 14 shows the mapping and path tracking results. Here, path tracking is represented by green marker. From the experimental results, it can be observed that the morphology of the robot has accommodated various drain structures, and handles the various terrain constraints inside the drain (Fig. 13). Furthermore, mapping and localization results (Fig. 14) ensure that the robot’s position can track in deep and unexplored drain environments with the help of SLAM generated map.

#### Real-time field trial

Evaluating the drain inspection algorithm with real-time video feed is the second and final phase of the field trial. The experiment was tested on the above-mentioned drain environment. The robot was operated in teleoperated mode and control by RF wireless communication module. The robot captured images are remotely analyzed by a drain inspection algorithm run on GPU enabled high-speed notebook. A total of 300 frames of images with various trash, bushes and silt objects within frame were captured during the real-time field trial and used for evaluation.

**Figure 15:**
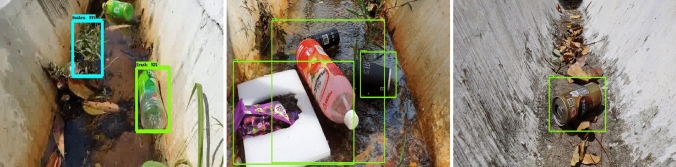
Trash detection on real time drain inspection field test (day).

**Figure 16:**
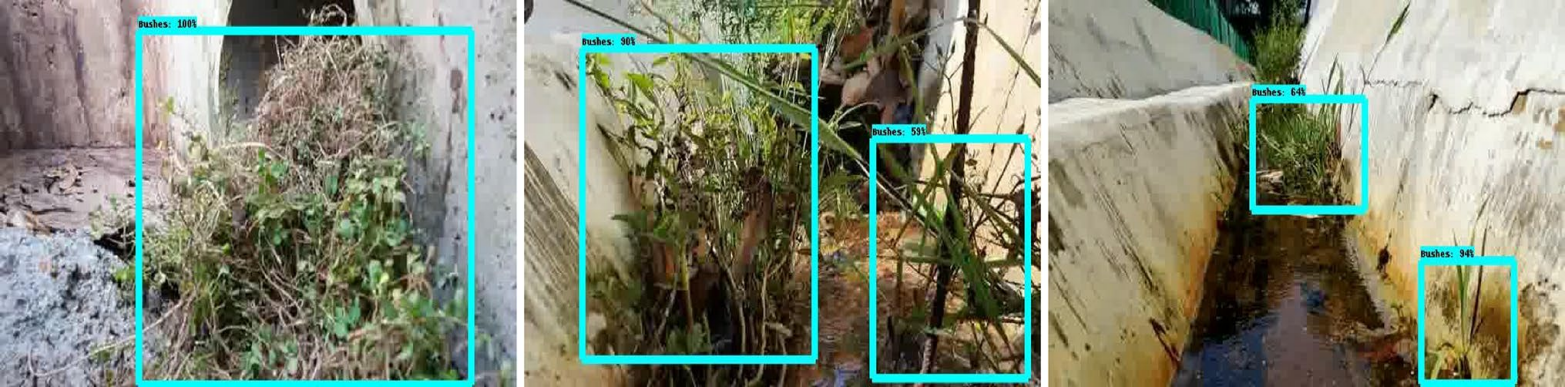
Bushes detection on real time drain inspection field test (day).

**Figure 17:**
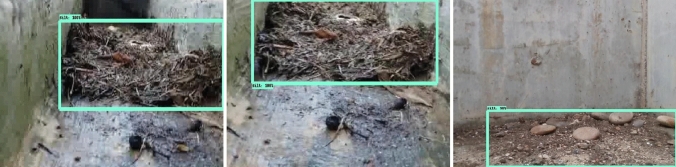
Silt detection on real time drain inspection field test (day).

**Figure 18:**
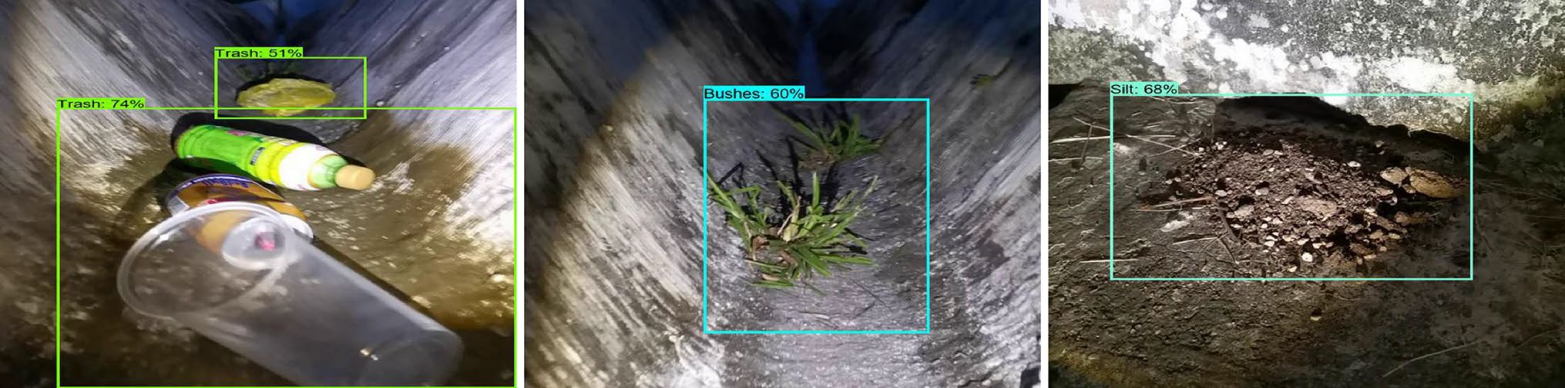
Real time drain inspection field test (night).

Figures 15, 16 and 17 show the drain inspection algorithm field test results where the open drain has running water, bushes are sprouted through cracks, and trashes floating on water. Similarly, the closed drain has the accumulated silt and trashes scattered and accumulated after a heavy rainfall. The field test was also done during the night time as seen in Fig. 18.

The detection algorithm has detected majority of trash, bushes, and accumulated silt on raptor robot captured images with a higher confidence level. In this two-drain experiment, the algorithm detects the bushes, trashes, and silt with an average of 88–92% confident level. Its bounding region is also accurate with respect to ground truth.

Furthermore, statistical measures have been performed for estimating the robustness of the drain inspection algorithm. For each individual class, 100 images were chosen and used for statistical measures. The images are chosen with a mix of both unseen offline and real-time field-collected images. Table 3 shows the statistical measures result of the SSD Inception v3 framework.

**Table 3:** Statistical measures results of drain inspection algorithm.

Weather condition	Class	Precision	Recall	F1	Accuracy	Average accuracy
Day	Bushes	94.34	94.81	94.57	95.25	91.42
	Trash	90.57	89.87	90.22	90.19	
	Silt	88.95	88.74	88.84	88.82	
Night	Bushes	92.94	92.01	92.48	93.47	90.18
	Trash	89.70	89.89	89.79	88.93	
	Silt	88.48	87.88	88.18	88.13	

The statistical measure indicates that the algorithm has detected the drain blocking objects with $91.42\%$ accuracy for combined test images (offline and real time collected drain inspection images) for day time and an accuracy of $90.18\%$ is achieved during the night time (with light source to illuminate path and objects). A small drop-off of accuracy of $1.24\%$ between day time and night time is reported. Further, the model miss rate is $4\%$ for offline test and $7\%$ for real time test images. The higher miss detection in real time is attributed to object occlusion, blurring due to jerk in robot navigation when moving on uneven surface, shadows, and varying lighting conditions. The research confirms that environmental influences such as varying lighting conditions and shadows have no significant impact on the suggested solution.

### Comparison analysis

This section examine the performance of SSD inception framework with baseline methods SSD MobileNet v2 and SSD VGG16 and also perform the comparison analysis with other state-of-the-art object detection methods including Yolo v3 and Inception variants. The comparison analysis was performed with mixed of real time field images and web collected images with a total of 100 images from each class. The detection frameworks were trained using a transfer learning scheme along with our custom drain inspection data set. Table 4 shows the comparison between the proposed framework, baseline methods and the other object detection frameworks along with computation time.

The results indicate that the proposed framework and the various SSD Inception versions have good detection accuracy compared with Yolo v3 and the two baseline methods. The key issue of Yolo v3 and the two baseline methods is the miss detection and false classification of objects. These two factors affect the overall performance of the two baseline methods and Yolo v3 framework. In computation cost analysis, Yolo v3 (31 FPS) and SSD MobileNet v2 (23 FPS) took lower execution time than SSD Inception v3 (18 FPS), SSD VGG16 (14 FPS), SSD Inception v4 (9 FPS) and SSD Inception ResNet v2 (7 FPS). Due to dense CNN layers, the various SSD Inception versions and SSD VGG16 computation time are quite high comparatively. However, this is does not heavily affect the performance of the real-time drain inspection task. It can be overcome by upgrading the computing hardware. In this analysis, we observe that SSD Inception v3 model strikes a balance between detection accuracy and computation time. Hence, SSD Inception v3 model is chosen as the optimal framework for the drain inspection task.

**Table 4:** Comparison analysis with baseline method and other object detection framework.

Model	Precision	Recall	F1	Accuracy	Detection speed (FPS)$^{2}$
SSD with MobileNet v2 (baseline)	86.45	85.92	86.18	86.38	23
SSD with VGG16 (baseline)	88.76	88.06	88.41	88.39	14
Yolo v3	85.22	84.24	84.73	85.66	31
**SSD with Inception v3**$^{1}$	**91.28**	**91.14**	**91.21**	**91.42**	**18**
SSD with Inception v4	91.20	91.93	91.56	91.58	9
SSD with Inception ResNet v2	91.26	92.01	91.64	91.55	7

${}^{1}$The bolded model is the framework used for real-time Raptor test.

${}^{2}$This refers to the inference time (ms) required by the respective model for one image converted into frame per second (FPS) metric, all models were tested on the same hardware and a total of 300 images (100 for each class) during the comparison analysis.

### Comparison with existing work

This section elaborates the comparative analysis of the proposed algorithm with existing drain inspection framework reported in literature based on inspection tool and inspection algorithms. Table 5 states the accuracy of various inspection models and algorithms based on some similar classes.

In Table 5^2–4,43^, uses the CCTV as inspection tool and applying different DL framework namely Resnet-TL, four layer custom CNN, five layer custom CNN and Modified ZF to automate the drain inspection. Here, Resnet-TL^2^, four^43^ and five layer CNN^4^ are classifier framework which scored an average precision of 85%, 87.7% and 78% (accuracy) for drain inspection tasks, respectively. Modified ZF^3^ is an object detector framework and uses Faster RCNN as the detector head. The implementation scored an average precision of 83% across four different classes; root, crack, infiltration and deposit.

In contrast with our implementation the above schemes’ classification and detection scores are quite low, however, the implementations cannot be compared directly with our work. Since their datasets, CNN topology and training parameters are totally different. Further, deploying and maintenance of CCTV for long range drain networks is a challenging task. More cameras are required to cover a more extensive area of the drainage. This will increases the cost of CCTV drain inspection system. The proposed framework utilises real-time inspection in comparisons to the above schemes which utilises offline inspection, this difference is reflected in the choice of algorithm used. Further, compared to Cheng et al. utilisation of Faster RCNN the proposed framework opted for a faster and lighter object detection framework, SSD.

**Table 5:** Comparison analysis with existing object detection framework.

Case studies	Inspection type	Algorithm	Classes	Precision
Tennakoon et al.^2^	Offline CCTV	Resnet-TL	5	85.00
Kumar et al.^43^	Offline CCTV	4 layer CNN	3	87.7
Moradi et al.^4^	Offline CCTV	5 layer CNN	1	$78.00^{2}$
Cheng et al.^3^	Offline CCTV	Modified ZF	4	83.0
**Proposed framework**	**Real-time with Raptor**	**SSD with Inception v3**$^{1}$	**3**	**91.28**

${}^{1}$The bolded model is the our proposed framework used for real-time Raptor test.

${}^{2}$The authors only reported the accuracy score.

**Table 6:** Comparison with existing drain inspection robots.

	Tarantula	MAKRO	KURT	PIRAT	Raptor
Morphology	Reconfigurable	Fixed-shape	Fixed-shape	Fixed-shape	Reconfigurable
Weight (kg)	20	30	–	–	2.45
Dimension (m)	1.2	1.6	$0.38\times 0.28\times 0.30$	–	$0.39\times 0.35\times 0.29$
Speed (m/s)	–	0.3	0.20	0.35	0.22

Table 6 shows the comparison analysis of current robotic platform with existing drain inspection platforms. Here, robots KURT, PIRAT, and MAKRO has adopted fixed-morphology and designed only for sewer pipe inspection with small diameter. Whereas, Tarantula is the reconfigurable drain inspection robot. However, the robot has high cost, complex mechanical design and heavy weight due to many actuators. Moreover, its maneuverability is not stable in some drain segments^16^. On the other hand, our in-house developed robot Raptor is lighter and has a simplistic design approach. Also, its three modes of reconfiguration provides good maneuverability in varied terrain condition. This design consideration is the core contribution of the proposed design with respect to the state-of-the-art.

## Conclusion

Remote drain inspection framework was proposed using Deep Learning based object detection framework and our in-house developed self-reconfigurable robot Raptor. SSD Inception v3 object detection module was trained for drain inspection tasks with common drain-blocking objects. The model was trained by a transfer learning scheme and used the pre-trained weights. The efficiency of the remote drain inspection framework was assessed 100 meters to 300 meters in three different drains including closed drain, semi-closed drain, and open-drain. The experimental results proved that our in-house developed robot maneuverability was stable and its mapping and localization is more precise in complex rugged drain terrain. The efficiency of the drain inspection algorithm was examined in two phases: offline test and real-time field trial using drain images captured by Raptor. Standard performance metrics including accuracy, precision, recall, and F1 measures were used to assess the drain inspection algorithm. The performance metrics results show that the drain inspection algorithm has detected the drain blocking objects, including bushes, trashes, and silt, with 91% detection accuracy. Further, comparison analysis was performed with other object detection frameworks, existing object detection framework and existing drain inspection platforms. The analysis results show that the various SSD inception versions and SSD VGG16 models have higher detection accuracy than SSD MobileNet and Yolo v3 framework. However, SSD MobileNet and Yolo v3 framework is capable of lower inference time. In this comparison, SSD Inception v3 model strikes the balance between detection accuracy and computation time. Its detection accuracy and inference speed outperforms SSD VGG16. All the analysis results ensure that our proposed framework can perform drain inspection tasks and have the capability to identify the drain blocking objects in closed, semi-closed, and fully opened extended drains in varying lighting conditions and weather conditions.
